# Left Ventricle Pseudoaneurysm: Contribution of Multimodality Imaging to the Diagnosis

**DOI:** 10.1155/2014/531929

**Published:** 2014-07-21

**Authors:** Ellenga Mbolla Bertrand Fikahem, Okemba-Okombi Franck Hardain, Mongo-Ngamami Flore Solange, Kouala Landa Christian Michel, Gombet Thierry Raoul, Kimbally-Kaky Suzy-Gisèle

**Affiliations:** ^1^Department of Cardiology, University Hospital of Brazzaville, P.O. Box 32, Brazzaville, Democratic Republic of Congo; ^2^Department of Medicine, Faculty of Health Science, Marien Ngouabi University, P.O. Box 2672, Brazzaville, Democratic Republic of Congo; ^3^Department of Pneumology, University Hospital of Brazzaville, P.O. Box 32, Brazzaville, Democratic Republic of Congo

## Abstract

The left ventricle pseudoaneurysm is an anomaly of the left ventricle and is severed and joined with a pocket look. There may be secondary to a myocardial infarction, trauma, or surgical procedure. Sometimes the cause is not found. Complications are heart failure, arrhythmias, vascular embolism, and sudden death. The treatment is surgical only. The authors report the case of a black patient of 64 years old, without medical history, had seen to a deformation of the cardiac shadow in radiography. The left ventricle pseudoaneurysm and in situ thrombus are visualized in echocardiography and CT scan. The patient is waiting for heart surgery.

## 1. Introduction

The left ventricle pseudoaneurysm (LVPA) is a complication related to the rupture of the free wall of left ventricle, which communicates through a defect with newly formed saccular pocket [1]. It is often secondary to a myocardial infarction, chest trauma, and a surgical or instrumental cardiac procedure [2–5]. Sometimes LVPA is discovered during a complication, the etiology being unseen [4, 6]. This is an unexpected anomaly that may cause sudden cardiac death [6, 7]. The others complications are heart failure, arrhythmias, and embolism [8]. In all cases, the surgical treatment is required [7]. This anomaly is rare in Africa. Six cases have been collected 18 years ago in Tunisia [7].

In this clinical case, the authors report a LVPA discovered incidentally.

## 2. Observation

**Figure 1:**
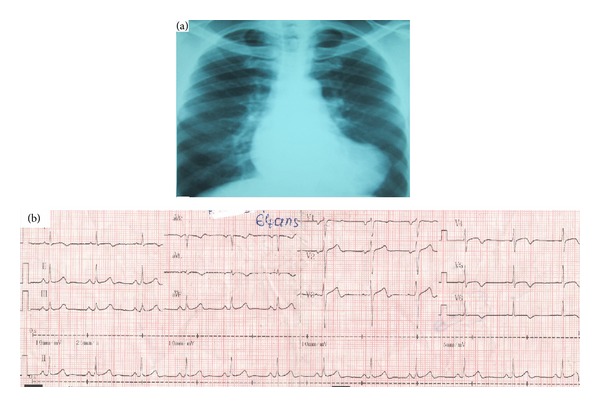
X-ray and electrocardiogram of this patient. (a) Front chest radiography, showing the deformation of the lower arc of left heart; (b) ECG was in sinus rhythm and objectified negative and symmetrical T waves in D1, aVL, V4, V5, and V6.

A 64-year-old black man, retired teacher, resident at Brazzaville (Republic of Congo), had consulted in pneumology examination for cough, mucopurulent sputum, and low-grade fever. The usual blood tests were unremarkable. The patient was treated with antibiotics and mucolytic for the diagnosis of acute bronchitis. The evolution was unremarkable. However, a chest radiograph had objectified an angulation of the lower left arc of the heart (Figure 1(a)); this was the reason for cardiology consultation.

Family and private medical history was not particular.

The patient was asymptomatic and in a good condition. His measurements were weight 70 Kg, height 1.76 m, and BMI 22.3. In cardiac examination, the heart rate was 95 beats per minute, with no murmur cardiac in auscultation; systolic blood pressure was 132 mmHg and 74 mmHg for diastolic blood pressure.

In blood chemistry, the hemoglobin was 15.2 g/dL, the blood sedimentation rate was 13 mm in 1st hour, the creatinine was 12.4 mg/L, and the glycaemia was 1.04 g/L. The other analysis suggests that transaminases, troponin, CPK-MB, blood lipids, were normal.

**Figure 2:**
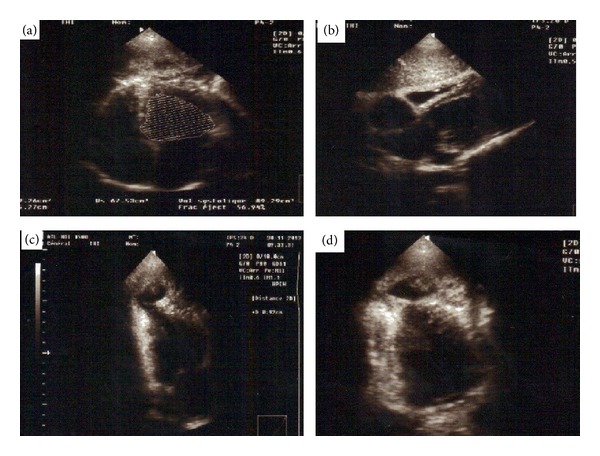
Cardiac ultrasound findings. (a) Calculation of ejection fraction from 57% in Simpson 4 cavities. (b) Cutting subcostal objectifying the left ventricle and the pseudoaneurysm with no notion of communication. ((c) and (d)) Apical view: collar diameter was 9 mm, and presence of a heterogeneous structure suggestive of thrombus lining the pseudoaneurysm was noted.

**Figure 3:**
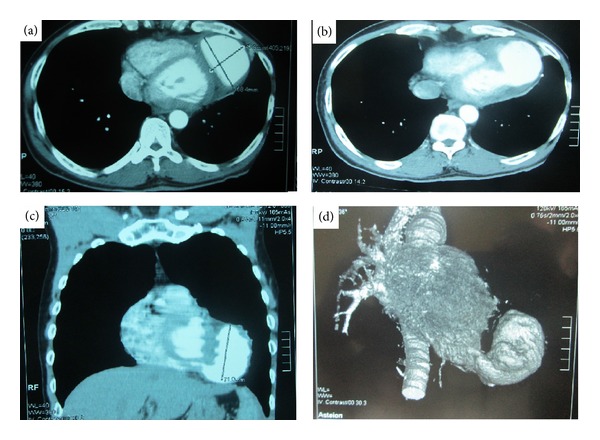
Chest CT scan imaging. (a) Cross-section showing the diameters of the pseudoaneurysm (45.9 mm × 68.4 mm). (b) Visualization of the neck between the left ventricle and the false aneurysm. (c) shows a sagittal diameter of 71 mm. (d) 3D reconstruction of the left ventricle communicating with the pseudoaneurysm.

The EKG in sinus rhythm had objectified negative T waves in the lateral area (Figure 1(b)). In echocardiography, heart dimensions were normal. The ejection fraction in Simpson four cavities was 57% (Figure 2(a)). We visualized a neocavity connected with the apex of left ventricle (Figure 2(b)) by a collar of 0.92 cm of diameter (Figure 2(c)). These aspects suggested a pseudoaneurysm. In addition, a poorly limited rounded mass was hyperechoic and pendant at the bottom of pseudoaneurysm, suggesting a thrombus (Figure 2(d)). The precise boundaries of the pocket could not be specified in ultrasound apical and subcostal. The thoracic CT scan with injection of contrast material had showed in cross-sectional (Figure 3(a)) the dimensions of LVPA: 45.9 mm × 68.4 mm. In the same section, a communication between the pseudoaneurysm and left ventricle is noted (Figure 3(b)). In front section (Figure 3(c)), the longest diameter of LVPA was 71 mm. A reconstruction of LVPA was performed in 3D imaging (Figure 3(d)).

The treatment included atenolol and acenocoumarol. The international normalized ratio (INR) was 3.2 at last medical checkup. Invasive cardiac investigation (coronary angiography) and cardiac surgery are not feasible in Congo. For this reason, the medical evacuation abroad is required for full support and to determine the aetiology.

## 3. Discussion

The LVPA is an anomaly rarely described in the literature [4]. Few cases have been reported in North Africa and sub-Saharan Africa [5, 7, 9]. Before the advent of ultrasound and CT scan, only angiography allowed the diagnosis [7]. This anomaly is secondary to myocardial infarction or cardiac trauma [3, 10]. Therefore, an array of heart failure or chest pain is the reason of consultation. Sometimes, the event goes unnoticed and LVPA is discovered long later [6].

The diagnosis of LVPA is placed in the imaging. The deformation of the cardiac shadow is noted in standard radiography [9]. However, the heart may have a normal appearance [9]. Echocardiography leads to diagnosis, objectifying communication between the left ventricle and the pocket [8], as in the case we described. The delimitation of contours can be complicated by the configuration of the pocket. Indeed, ultrasound appeared limited in our case, because the pseudoaneurysm communicated with the left ventricle at the tip, and went back under the ribs forward. Transesophageal ultrasound would have a better analysis of the pseudoaneurysm. CT scan has proven to be an indispensable tool in our case, to define the limits of pseudoaneurysm. Other authors have also used this exploration for a comprehensive assessment of the lesions [8].

Aside from the mediastinal rupture and sudden death, the other complications of LVPA are heart failure, arrhythmias, and vascular embolism [6, 10]. In the absence of major complications, the treatment is comprised of beta-blockers and/or anticoagulants. In our case, an indication of acenocoumarol was justified after viewing an intra-anevrismal thrombus in echocardiography [9].

The cure is the open heart surgery in which a resection of the bag is made [7]. Surgery is indicated even in asymptomatic patients as outlined by Mecheche et al. [7]. Surgical treatment can prevent sudden death. In patients with high surgical risk, an instrumental closure may be achieved [5]. An indication of aneurysmectomy was accepted in our case. The heart surgery was not available in Congo; the complete treatment will be performed abroad. The postoperative course is usually simple. After cardiac surgery, there is no recurrence of pseudoaneurysm.

Through this clinical case, we have shown the limits of treatment despite an accurate diagnosis in our country and in most countries of sub-Saharan Africa. In absence of cardiac surgery, the patient will be exposed to a sudden death.

## 4. Conclusion

The pseudoaneurysm of left ventricle is an anomaly shortly described. It is rarely asymptomatic idiopathic and puts the patients at risk of sudden death. Diagnosis involves several imaging modalities that can guide the surgeon.
